# Bactrian Camel Milk: Chemical Composition, Bioactivities, Processing Techniques, and Economic Potential in China

**DOI:** 10.3390/molecules29194680

**Published:** 2024-10-02

**Authors:** Shamila Seyiti, Abulimiti Kelimu, Gulinaer Yusufu

**Affiliations:** 1 School of Economics and Management, Xinjiang University, Shengli Road 666, Urumqi 830046, China; gulinaeryusufu@xju.edu.cn; 2 College of Food Science and Pharmacy, Xinjiang Agricultural University, Nongda East Road 311, Urumqi 830052, China

**Keywords:** Bactrian camel, camel milk, chemical composition, bioactivities, economic potential

## Abstract

Bactrian camel (BC) milk has gained increasing attention due to its unique nutritional profile and potential bioactivities. This comprehensive review explores the chemical composition, bioactivities, processing techniques, and economic potential of BC milk in China. The distinctive chemical composition of BC milk, including protein, lipid, carbohydrate, vitamin, and mineral content, is discussed, emphasizing its differences from other mammalian milk. The review highlights the various bioactivities of BC milk, such as anti-inflammatory, antidiabetic, lipid-lowering, and anticancer properties, as well as its modulatory effects on intestinal microbiota. The technological properties of BC milk, focusing on its heat stability, coagulation behavior, and potential for product development, are examined. The review also addresses current processing techniques and their impact on milk quality. Finally, the economic potential and future perspectives of BC milk in China are evaluated. This review provides valuable insights into the multifaceted aspects of BC milk, serving as a foundation for future research and development in this emerging field. The motivation for this review stems from the growing interest in BC milk as a functional food and the need for a comprehensive understanding of its properties, applications, and market potential to guide future research and industry development.

## 1. Introduction

Camels play a wide variety of roles, providing milk, meat, long-distance transportation, and other commodities for people living in extremely arid desert areas. Unlike other common livestock, camels possess an unparalleled ability to thrive and provide services under extreme conditions, such as food and water scarcity and high temperatures. However, with the advancement of science and technology, the social roles of camels have transformed into a new era, continuing to contribute to the livelihood of desert people by providing more value-added products, such as milk [1].

The Camelidae family is characterized by two defined subfamilies: *Camelinae* (Old World camelids) and *Laminae* (New World camelids). Old World camelids, which make up the majority of the camel population, include two domesticated and one wild species: the Dromedary camel (DC, *Camelus dromedarius*, one-humped), the BC (*Camelus bactrianus*, two-humped), and the endangered wild camel (*Camelus ferus*, two-humped). The DC, which inhabits hot and arid areas of the Middle East, Eastern and Northern Africa, Southwest Asia, and Australia, represents one of the most prevalent and widely reared species. On the other hand, BC has been recognized as an umbrella species for the fragile ecosystem of Central Asia and mostly occurs in the cooler desert regions of northwestern China, southern Russia, Mongolia, Kazakhstan, and Asia Minor [2]. It is a numerically inferior species, accounting for 10% of the total camel population [3]. As a lesser-known camel population, *Laminae* (New World camelids) are mainly distributed at high altitudes in South America and are generally divided into two wild species, guanaco (*Lama guanicoe*) and vicuña (*vicugna X*) and two domesticated species, llama (*Lama glama*) and alpaca (*Vicugna pacos*) [4].

In recent decades, the camel population in Africa has increased approximately twofold, while it has decreased gradually in Asia. This is because, in arid countries, extensive camel breeding remains the main agricultural activity due to its cost-effectiveness in terms of feed conversion, longer lactation period, and unique ability to adapt to harsh environments compared to other milk-producing mammals. In addition, as an integral part of their diet, camel milk occupies a pivotal position with respect to its role in the nutritional uptake of nomadic communities, as it is an excellent source of protein, fatty acids, minerals, and vitamins [5]. Camels also enable people in marginalized communities in certain geographical areas to make a living and support their families by selling camel products (milk, meat, and wool) and providing services in social activities such as tourism and leisure. In contrast, owing to the decreased necessity of utilizing camels for traditional purposes, such as transportation and agriculture, the number of camels reared in Asia has decreased gradually [6]. As reported by the Food and Agriculture Organization of the United Nations [7], the world camel population reached about 41.77 million in 2022.

Although camel milk has long been known for its excellent nutritional value and various bioactivities, it is consumed primarily by desert dwellers in its fresh state or after converting it into traditional fermented beverages through spontaneous fermentation. Over the past few decades, research on camel milk has increased, leading to several studies investigating its contents and functions [8,9]. Being widely regarded as a sustainable and promising livestock species for the development of health-beneficial foods or ingredients, camel milk consumption is gradually becoming popular among urban consumers due to China’s large population and rapid economic development. With the widening gap between supply and demand, alongside the rising price of camel milk, camel farming has evolved into a highly profitable business. Both the government and the private sector recognize that sustained increases in the prices of camel milk and its products could significantly enhance economic livelihoods and alleviate poverty in rural areas with harsh climates, provided that proper management and utilization are available. Given the economic and nutritional significance of camel milk, there has been a gradual increase in capital investment and large-scale commercial production on modern camel farms. Currently, camel milk products, including pasteurized milk, fermented beverages, and dehydrated products, are readily available on the market. While the individual components, physicochemical characteristics, bioactivities, and technological properties of DC milk have received increasing attention since it is the most widely reared species worldwide, BC, which is the predominantly reared species in Asian countries, is understudied in terms of the composition, bioactive components, and technological aspects of its milk, with very limited reviews available, especially in terms of bioactivities and technological aspects. Moreover, studies have shown that some components (protein, fatty acids, oligosaccharides, etc.) of BC milk are significantly different from those of DC milk [10,11]. From a technological perspective, the distinct composition of BC milk may influence its behavior during processing and product development. Given the unique characteristics of BC milk and the limited understanding of its functional and technological aspects, a comprehensive review of the available research findings is warranted. Such a review would not only shed light on the potential benefits and applications of BC milk but also contribute to the broader understanding of camel milk diversity and its potential for value-addition in the dairy industry.

Hence, the main purpose of this review is to summarize the fragmentary knowledge of BC milk, with an emphasis placed on the camel population and milk yield, composition, bioactivities, processing, and economic potential.

## 2. Methods

A systematic literature search was carried out to identify pertinent studies on Bactrian camel and camel milk. This comprehensive search involved various databases and official websites to ensure a thorough examination of the existing scientific literature. The sources utilized for identifying relevant research articles and reports included Google Scholar (https://scholar.google.com, accessed on 15 August 2024), Science Direct (www.sciencedirect.com, accessed on 10 August 2024), the American Chemical Society Database (https://pubs.acs.org, accessed on 25 July 2024), and the China National Knowledge Infrastructure (CNKI) Database (www.cnki.net, accessed on 15 August 2024). The search strategy was guided by specific keywords such as “camel milk” and “Bactrian camel milk”, chosen to capture a broad range of studies on the topic. The focus was primarily on research conducted in China on Bactrian camel milk in recent years.

The inclusion criteria for the literature encompassed studies that specifically addressed the nutritional, biochemical, or therapeutic properties of Bactrian camel milk, research articles, reviews, and reports published in peer-reviewed journals, and studies available in English or with an English abstract. The search was systematically conducted across the selected databases, and relevant articles were screened based on their titles and abstracts. Full texts of potentially relevant articles were then retrieved and reviewed in detail.

## 3. Camel Population

**Figure 1:**
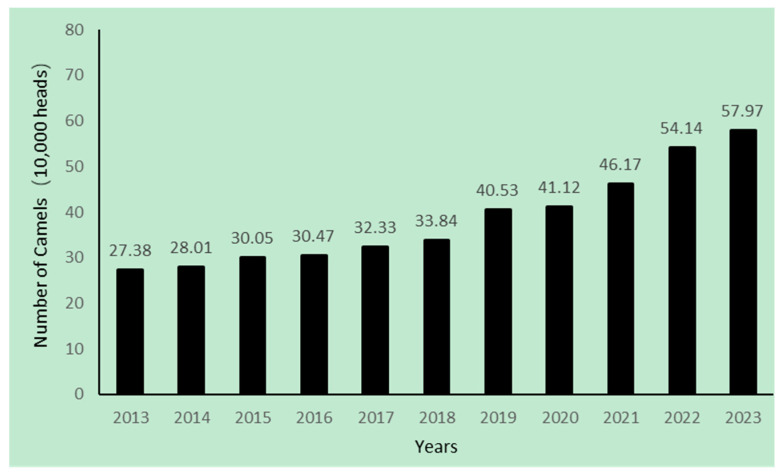
Change in number of camels from 2013 to 2023 (NBS of China 2023).

Throughout Chinese history, camels have played a crucial role in civilization. Due to their unique adaptations to diverse and extreme environments, camels became the primary means of caravan traffic along the Silk Road, facilitating long-distance migration in desert areas. Beyond their role as pack animals, camels provide economically deprived communities with milk, meat, and wool. Most camels in China are Bactrian, mainly distributed in mountainous and marginal regions, where dairy camel farming is increasingly viewed as a profitable business. According to the National Bureau of Statistics, China’s camel population has grown at a compound annual growth rate of 10% from 2013 to 2023, reaching approximately 579,700 camels in 2023 (Figure 1). The majority are found in the provinces of Xinjiang (52.44%), Inner Mongolia (37.62%), Gansu (7.19%), and Qinghai (2.48%) [12]. The “National List of Animal Genetic Resources and Varieties” (2021) characterizes China’s camel genetic resources by five defined breeds: Alxa BC and Sonid BC from Inner Mongolia, Tarim BC and Junggar BC from Xinjiang, and Qinghai BC [13].

## 4. Milk Yield

Generally, in terms of milk production, camels are considered less productive, with BC being even less productive than DC. However, camel milk, especially from BC, is richer in dry matter, including fat, protein, and lactose [14,15]. Nurseitova et al. (2014) reported no significant difference in milk yield between BC (2.9 L/d) and DC (3.2 L/d) reared under the same conditions, although BC milk had higher fat and dry matter content [16]. These conflicting results could be attributed to varying farming conditions [15]. According to a recent study, the milk production of Bactrian camels in China varies between 1470.18  ±  9.75 kg and 978.34  ±  3.80 kg over a period of 300 days [17].

## 5. Physicochemical Properties of Camel Milk

Camel milk, characterized by its opacity and white color due to its finely dispersed fat, possesses a unique combination of sweetness, sharpness, and occasional saltiness. Agitation of the milk leads to the easy formation of froth. Throughout the lactation cycle, the physicochemical properties of camel milk undergo significant changes. Specifically, the relative density hovers between 1.028 and 1.045; the viscosity ranges from 6.79 to 24.66 mPa·s; the pH varies between 6.35 and 6.79; the acidity falls within 0.17–0.24%; the thörner degree, a measure of titratable acidity, ranges from 19.98 to 20.71; and the conductivity is measured at 0.38–0.57 mS/cm. As the dry matter content decreases during lactation, both the density and viscosity of camel milk gradually decrease, while the other physicochemical properties tend to remain relatively constant [1,18,19]. The composition of camel milk, and consequently its flavor and other attributes, is influenced by several factors, with fodder type and water availability being particularly significant [20]. In terms of shelf life, raw camel milk outperforms bovine milk, remaining stable for an extended period at 30 °C and maintaining its quality for 2–3 days at room temperature without any visible changes. When refrigerated at 4 °C, it can be kept for more than three months without showing any signs of deterioration.

## 6. Chemical Composition

BC milk, like other animal milks, provides a wide array of nutrients, such as proteins, lipids, lactose, and other essential components, making it a valuable source of nutrition for a balanced diet. However, BC milk is distinct from other mammalian milks in terms of certain chemical components, including individual proteins, fatty acids, and various bioactive ingredients that may offer health-promoting benefits [9,21]. Understanding the nutritional quality of milk is critical for its application and processing. Over the past two decades, considerable efforts have been made to unveil the compositional characteristics of this unique food resource (Table 1). Despite the fact that the milk yield of BC is lower than that of DC, BC milk is richer in protein, fat, and total solids [14,22], which makes it superior to DC milk in terms of nutritional value and suitability for processing. The composition of camel milk is influenced by several factors, including seasonal variations, geographical origins, camelid species, nutritional conditions, breed, stage of lactation, age, and parity [17,19,23,24,25].

### 6.1. Camel Milk Protein

Camel milk is increasingly recognized as a valuable source of proteins that exhibit a wide range of abundances and varieties. These proteins can be broadly categorized into three primary groups: casein (CN), whey protein (WP), and milk fat globule membrane (MFGM) proteins. Among these, casein components such as α-CN, β-CN, and κ-CN [38], along with whey proteins such as α-lactalbumin (LA), serum albumin (SA), lactoferrin (LF), immunoglobulins, and peptidoglycan recognition protein, are the most prominent [11]. MFGM proteins also play a significant role in the overall protein composition of camel milk [10,39]. Few studies have explored the relative distribution of milk proteins in different breeds of camels, such as BC and DC. For instance, research has shown significant variations in protein composition between BC and DC milk [11,40]. Using peptidomics techniques, a study identified 622 parent proteins from 8393 peptides in camel milk. Among these proteins, 208 were from DC, and 464 were from BC. After filtering, 4464 endogenous peptides were quantified, 459 of which were common to both breeds. Notably, 170 peptides derived from 27 proteins exhibited significant differences between the two types of camel milk. For example, osteopontin and lactoperoxidase were upregulated in DC milk, whereas butyophilin subfamily member A1, perilipin, and fatty acid synthase were upregulated in BC milk [40]. Compared to other animals, camel milk exhibits distinct proportions of protein components, showing similarities primarily with human milk [41]. Camel milk proteins also demonstrate enhanced digestibility and unique peptide profiles [9], underscoring their potential suitability for infant formula applications.

#### 6.1.1. Casein

Camel milk is known for its nutritional value and therapeutic effects, largely due to its bioactive components, including proteins and peptides. Casein, the main protein complex in BC milk, exists in a micellar form distinct from that in ruminant milk [42]. This heterogeneous protein is composed of αs_1_-CN, αs_2_-CN, β-CN, and κ-CN, which vary in amino acid sequence and posttranslational modifications. Although studies have reported different relative proportions of these proteins [39,43,44], they have consistently indicated that β-CN is the most abundant casein fraction. A low level of β-CN phosphorylation is associated with the formation of a softer and less cohesive curd, facilitating easier digestion. The abundance of β-CN in camel milk underscores its superior digestibility and hypoallergenic properties, making it particularly beneficial for neonatal infants [9]. Future research on camel milk casein should focus on detailed structural and functional studies to fully understand its unique properties. Exploring its potential in infant nutrition, particularly for those with allergies, is crucial given its superior digestibility and hypoallergenic nature.

#### 6.1.2. Whey Proteins

Renowned for its exceptional nutritional profile, camel milk is abundant in whey proteins and exhibits significant variations in both the quantity and distinctive attributes of its proteins compared to those of cow’s milk [10]. Among the diverse array of whey proteins detected in camel milk, the predominant ones include serum albumin (SA), α-lactalbumin (LA), lactoferrin (LF), lactoperoxidase (LPO), and glycosylation-dependent cell adhesion molecule 1 (GLYCAM1). Notably, the levels of SA, LF, and GLYCAM1 are significantly higher in camel milk than in bovine and goat milk [10]. The structural characteristics of serum albumin (SA) in camel milk include a notably high alpha-helix content, minimal beta-fold structures, and an irregular coil configuration, which are crucial for its functionality, stability, and intermolecular interactions [45]. Yang et al. [46] reported that LA, comprising 123 amino acid residues with a molecular mass of 14.6 kDa, is the most abundant whey protein in BC milk. This contrasts with bovine LA, which contains 142 amino acid residues and has a molecular mass of 16.2 kDa [47]. To elucidate the genetic and biological diversity across animal species, researchers have identified whey acidic protein and quinone oxidoreductase as distinctive traits characterizing camel milk [46]. Camel milk whey protein also contains naturally occurring heavy-chain antibodies (HCAbs), which have rehabilitative effects on infection and immunity. A study revealed that the IgG1 fraction within BC milk comprises molecules with heavy chains weighing 50 kDa and light chains weighing 36 kDa. In contrast, the IgG2 and IgG3 fractions lack light chains and consist solely of heavy chains with molecular weights of 45 kDa and 43 kDa, respectively. The abundance of heavy chains in the IgG2 and IgG3 fractions notably exceeds that in the IgG1 fraction [48].

#### 6.1.3. Amino Acids

In terms of amino acid composition, researchers have investigated the amino acid profile of BC milk throughout the lactation period. The findings revealed the presence of 17 amino acids, excluding tryptophan. Essential amino acids (EAAs) accounted for approximately 43.52–43.87% of the total amino acids (TAAs) during lactation, surpassing the recommended standards of the FAO/WHO. The ratios of EAA/TAA and EAA/NEAA (nonessential amino acids) were above 40% and 75%, respectively. Glutamic acid and leucine were the most abundant amino acids, followed by proline and lysine, with cysteine being the least abundant. The abundance of amino acids varied with the lactation stage, peaking in early lactation and gradually decreasing as lactation progressed [19]. Amino acid analysis revealed that the total amino acids, total essential amino acids, total drug-effective amino acids, and nearly all amino acids in Inner Mongolian BC milk were significantly higher than those in bovine milk [49] This enhanced amino acid profile further highlights the nutritional superiority of camel milk over bovine milk.

### 6.2. Lipids

#### 6.2.1. Lipid Composition

Camel milk has a distinctive lipid profile that differentiates it from other dairy sources and the lipid composition of BC milk in China also exhibits a diverse array of lipid classes. Zhao et al. [50] compared the lipid profiles of camel milk to those of other types of milk, identifying four main lipid classes—glycerolipids (GLs), glycerophospholipids (GPs), sphingolipids (SPs), and sterol lipids (SLs)—and 20 subclasses—triglycerides (TGs) and diglycerides (DGs). One study revealed that human milk contains 909 distinct lipids, while cow, goat, and camel milk contain 903, 918, and 907 distinct lipids, respectively. Notably, camel and human milk share the most similarities, with only 87 differential lipids, indicating a closer lipid profile. In camel milk, TGs constitute 90.08% of the lipid content, followed by phosphatidylcholine (PC, 4.28%), sphingomyelin (SM, 3.11%), phosphatidylethanolamine (PE, 1.26%), phosphatidylserine (PS, 0.38%), and other phospholipids (PLs). This study also underscores the similarities between human and camel milk lipid compositions, which could inform the development of better infant formulas. A greater proportion of functional TGs and PLs in camel milk could enhance the nutritional profile of infant formula, making it a potentially superior base [51].

#### 6.2.2. Polar Lipids

Polar lipids, such as phospholipids and sphingolipids, are vital components of biological membranes and have potential health-promoting properties. They play significant physiological roles, including treating dyslipidemia, reducing inflammation, improving cardiovascular health, and promoting intestinal and neural development [52,53]. Recent research in China has focused on the polar lipids in BC milk, examining their unique composition, potential health benefits, and methods for quality assessment. A comprehensive study used ultra-performance liquid chromatography–tandem mass spectrometry to isolate and analyze these lipids, identifying a total of 333 polar lipids [52]. Consistent with previous research [50], these polar lipids primarily included GPs and SPs. Camel milk showed high levels of PE, SM, and PC. The lipid profile included 77 PEs, 60 PCs, 28 PIs, 20 PSs, 4 phosphatidylglycerol (PGs), 3 phosphatidic acids (PAs), 14 lysophosphatidylethanolamines (LPEs), 13 lysophosphatidylcholines (LPCs), 1 lysophosphatidylinositol (LPI), 59 SMs, and 54 ceramides (Cers) [52]. This detailed profiling underscores the nutritional and functional significance of the complex lipid composition of camel milk. Researchers have employed advanced analytical methods to study the lipid composition of BC milk. A notable study used ultra-performance supercritical fluid chromatography (UPSFC) combined with quadrupole time-of-flight mass spectrometry (Q-TOF-MS) for high-throughput analysis of polar lipids in milk samples. This technique identified a wide variety of lipid species, including PE, PC, and SM, present in all the samples. A previous study revealed that the phospholipids in camel milk are predominantly in the order of PE, followed by SM and then PC [54]. Furthermore, a study comparing the phospholipid (PL) profiles of human colostrum and colostrum from six dairy animals (mare, camel, goat, cow, yak, and buffalo) revealed that camel colostrum was most similar to human colostrum [55]. These findings highlight the unique properties and potential health benefits of camel milk lipids, suggesting further research to explore their applications in nutrition and medicine.

#### 6.2.3. Fatty Acid Profile

The fatty acid composition of BC milk has been extensively studied and compared with that of human and other milks, revealing a unique and nutritionally significant profile (Table 2). Camel milk fat contains a wide range of fatty acids, including saturated fatty acids (SFAs), monounsaturated fatty acids (MUFAs), and polyunsaturated fatty acids (PUFAs). The composition of fatty acids in BC milk has been a subject of interest in recent studies [56,57,58,59]. A study by Zou et al. [51] revealed a distinct milk lipid composition characterized by the absence of short-chain saturated fatty acids (SC-SFAs) and a limited presence of medium-chain saturated fatty acids (MC-SFAs). The levels of long-chain saturated fatty acids (LC-SFAs) in camel milk fat are significantly greater than those in human milk fat (HMF), which is attributed to elevated levels of myristic acid (C14:0) and stearic acid (C18:0). The monounsaturated fatty acid (MUFA) content in camel milk fat (41.00 ± 1.20%) is comparable to that in HMF (36.90 ± 5.95%) due to high concentrations of palmitoleic acid (C16:1) and oleic acid (C18:1). In contrast, the PUFA content in camel milk lipids (3.31 ± 0.27%) was significantly lower than that in HMF (10.57 ± 4.96%), primarily due to the higher linoleic acid (C18:2 ω-6) content in HMF. The presence of SFAs at the sn-2 position is crucial for infant digestion, absorption, and metabolism. Despite lower SFA and PUFA levels at the sn-2 position in camel milk compared to HMF, MUFA levels are significantly greater, driven by substantial amounts of C16:1 ω-7 (10.72 ± 0.27%) and C18:1 ω-9 (27.63 ± 0.66%). The SFA and LC-SFA contents in camel milk phospholipids are considerably lower than those in HMF and other mammalian milks; however, MUFA (32.95 ± 1.44%) and PUFA (22.42 ± 1.14%) levels are significantly elevated.

In examining the effects of the lactation period on camel milk fatty acids, studies have evaluated the differences in fatty acid composition of BC milk samples collected from Inner Mongolia during lactation. They found that 23 fatty acids were detectable in BC milk, predominantly oleic (C18:1n9c), palmitic (C16:0), myristic (C14:0), and stearic (C18:0) fatty acids, which constituted 81.73% of the total fatty acids. Saturated fatty acids (SFAs) increased significantly with advanced lactation, while MUFAs and PUFAs showed the opposite trend. SFAs ranged from 50.71% to 58.84%, mainly C16:0 (29.8%), C14:0 (21.05%), and C18:0 (17.12%). MUFAs, primarily oleic acid (C18:1n-9), made up 26.81–34.79%, and PUFAs included C18:2n-6 (2.62–3.17%) and C18:3n-3 (0.72–1.03%) [19,58].

Compared to DC milk, SC-SFAs (C6:0 and C8:0) were not detected in the lipids from either camel species. In DC milk, the predominant saturated fatty acids (SFAs) were myristic (C14:0; 10.55%), palmitic (C16:0; 27.9%), and stearic (C18:0; 12.99%). These values significantly differed from those in BC milk, which had different percentage of myristic (11.54%), palmitic (31.51%), and stearic (18.67%) fatty acids. Compared with DC, BC presented a greater SFA content, corroborating previous findings by Zou et al. [51]. The MUFA content in BC milk was lower than that in DC milk, which was attributable to the higher levels of C16:1 and C18:1 in the latter. The data also revealed that the SFA content at the sn-2 position in DC was lower than that in BC. For the sn-1/3 positions, the primary fatty acids in DC milk fat were myristic (10.05%), palmitic (22.02%), and stearic acids (16.06%), whereas in BC, they were 11.90%, 23.79%, and 22.49%, respectively [60]. Interestingly, the fatty acid profile of BC milk appears closer to that of human milk than to that of cow milk. Several studies have reported that the levels of essential fatty acids, linoleic acid (C18:2n-6), α-linolenic acid (C18:3n-3), and arachidonic acid in camel milk are similar to those in human milk and higher than those in cow milk [58].

Compared with bovine and human milk, BC milk has a greater content of odd-chain saturated fatty acids (OCSFAs) and branched-chain saturated fatty acids (BCSFAs) [61], which are important for perinatal nutrition and intestinal microflora colonization in neonates [62]. Trans-monounsaturated fatty acids (trans-MUFAs), which are reported to have detrimental effects on human health, are present in greater amounts (2.46 ± 0.13 g/100 g) than in bovine milk (1.71 ± 0.02 g/100 g). Although the total amount of cis-MUFAs in camel milk is lower, the content of palmitoleic acid (C16:1 cis-9) is significantly higher than that in human and bovine milk [63]. This research also revealed the fatty acid profile of the milk, identifying that the dominant phospholipid fatty acids are palmitic acid (16:0), which ranges between 5.40% and 12.11%; stearic acid (18:0), which ranges from 10.83% to 22.60%; oleic acid (18:1), which ranges from 25.02% to 33.85%; and linoleic acid (18:2), which constitutes between 7.42% and 22.05% of the fatty acid content. This comprehensive lipidomic profile of camel milk underscores its unique nutritional characteristics and potential health benefits, as detailed by Jiang and colleagues in their 2022 study [54].

There are several differences in PUFA content, but C18:2 cis-9,12, C18:3n-3 (ALA), C20:4n-6 (AA), C20:5n-3 (EPA), and C22:5n-3 (DPA) exist in relatively high amounts [55,56,57,58,59].

**Table 2:** Fatty acid composition of camel milk from different studies.

Camel Species	SFAs (%)	MUFAs (%)	PUFAs (%)	References
Junggar BC	53.66 ± 4.85	41.00 ± 1.20	5.21 ± 0.34	[51]
Junggar BC	63.83 ± 3.11	30.27 ± 2.17	2.33 ± 1.21	[59]
Alxa BC	64.1 ± 0.87	25.7 ± 0.38	8.10 ± 0.11	[58]
Alxa BC	57.88 ± 3.88	31.35 ± 2.84	2.64 ± 0.12	[64]
Junggar BC	51.9 ± 2.20	39.6 ± 2.40	8.46 ± 0.86	[63]
Alxa BC	53.56 ± 1.86	37.99 ± 2.31	3.23 ± 0.14	[19]
Junggar BC	62.82 ± 0.05	31.72 ± 0.20	4.86 ± 0.25	[65]

Abbreviations: BC, Bactrian camel; SFAs, saturated fatty acids; MUFAs, monounsaturated fatty acids; PUFAs, polyunsaturated fatty acids.

### 6.3. Milk Fat Globule Membrane Composition

#### 6.3.1. Lipids

Lipids are dispersed in milk in the form of spherical or globular droplets of varying sizes. These droplets are encapsulated by MFGM, which is secreted from the membrane of mammary epithelial cells [66]. The MFGM is a complex bilayer that plays a crucial role in preventing fat droplets from flocculating and coalescing in milk [67]. This membrane has a tripartite structure composed of various components, such as proteins, glycoproteins, enzymes, neutral lipids, polar lipids, and cholesterol [68]. This unique structural arrangement and compositional complexity not only have technological importance in milk processing [69,70,71] but also confer several biological functions. These functions are attributed to the presence of biologically important components, including polar lipids [51,72,73].

The composition of the milk fat globule membrane (MFGM) of camel milk is distinct from that of bovine milk. The total fat/MFGM and neutral fat/total fat ratios in camel milk are relatively similar to those in bovine milk. However, the polar fat/total fat ratio is significantly lower, while the phospholipid/polar fat ratio is markedly greater in camel MFGM than in bovine MFGM [74]. Phospholipids, which are natural emulsifying agents ensuring the stability of milk fat globules [75], constitute the main polar fat in camel MFGM, making up 94.31 ± 2.27% of the polar fat content [74]. These differences in MFGM composition highlight the unique nutritional and functional properties of camel milk, making it a subject of interest for further research and potential applications in the food and health industries.

#### 6.3.2. Proteins and Amino Acids

The milk fat globule membrane (MFGM) proteome has been extensively studied across various species and lactation stages, highlighting its nutraceutical properties and potential benefits for human health. Proteomic analyses have been conducted on MFGM proteins from different mammals, including bovine, goat, human and camel milk, revealing a wide range of proteins present in these milk fractions [10,39,42,73]. Comparative proteomic analyses have been performed to examine the MFGM protein compositions of different camel breeds, specifically BC and DC. These analyses identified 911 proteins common to both breeds, along with 136 proteins unique to BC and 86 proteins unique to DC, from a total of 1047 and 997 proteins identified in the MFGM fractions of BC and DC milk, respectively. Among these proteins, lactadherin, butyrophilin subfamily 1 member A1, xanthine dehydrogenase, perilipin, and glycosylation-dependent cell adhesion molecule 1 were identified as the five most abundant proteins in the MFGM fractions of both BC and DC [11]. In another study, Han et al. [10] identified a total of 1579 MFGM proteins across bovine, goat, and camel milk samples. The findings revealed that 966, 1105, and 813 MFGM proteins were detected in bovine, goat, and camel milk, respectively. Among these proteins, 395 MFGM proteins were common across all three milk types. Specifically, 24 MFGM proteins were uniquely shared between bovine and camel milk, while 64 proteins were shared between goat and camel milk. In contrast, 120, 219, and 330 MFGM proteins were unique to bovine, goat, and camel milk, respectively. The functional analysis indicated that these MFGM proteins shared similar functional annotations, predominantly in cellular processes, intracellular anatomical structures, and binding, according to Gene Ontology (GO) annotations. A recent study assessed the protein composition of MFGM samples from camel and cow milk using a label-free quantitative proteomics approach. In total, 1427 and 1751 MFGM proteins were identified in cow and camel milk samples, respectively. This analysis revealed 1067 proteins common to both samples, while 684 proteins were unique to camel milk and 360 were unique to cow milk. The predominant MFGM proteins in both camel and cow milk included butyrophilin (BTN), xanthine dehydrogenase/xanthine oxidase (XDH/XO), fatty acid-binding protein (FABP), and fatty acid synthase (FASN) [39]. Efforts to characterize camel MFGM composition have also shown that the total dry matter and individual components, excluding moisture, are lower in camel MFGM than in bovine MFGM. Essential amino acids (EAAs), nonessential amino acids (NEAAs), flavor amino acids (FAAs), and the sum of amino acids (AAs) are present in lower amounts in camel MFGM protein than in bovine MFGM protein. In total, 17 amino acids were identified in bovine MFGM, while only 15 amino acids were detected in camel MFGM. Notably, camel MFGM contained a significantly higher amount of phenylalanine (Phe) (2.75 ± 0.29%). Methionine (Met), tyrosine (Tyr), and proline (Pro) are absent from the camel MFGM protein, whereas only Met is absent from the bovine MFGM protein [74].

### 6.4. Oligosaccharides (OSs)

Oligosaccharides, often referred to as prebiotics, are long-chain carbohydrates that have gained significant attention due to their potential health benefits. BC milk has emerged as a promising source of these functional compounds, and recent years have seen extensive research into the composition [24,76] and potential applications of BC milk oligosaccharides.

One of the pioneering studies in this field was conducted by Shi et al. [77], who analyzed various OSs in milk from humans, cows, goats, sheep, and camels. They found that 30 OSs were identified in bovine milk, 42 in caprine milk, 32 in ovine milk, 34 in camel milk, and 35 in human milk. Notably, camel milk had the highest similarity to human milk in terms of the types of OSs present, sharing a total of 16 common OSs, more than any other animal milk studied. However, the concentrations of eight specific OSs in human milk were approximately six times greater than those in camel milk.

In a comprehensive study, Zhang et al. [78] investigated the variations in milk OSs across different lactation stages and species. They identified a total of 89, 97, 115, and 71 OSs in human, bovine, goat, and camel milk, respectively. The number of common OSs shared between camel and human milk was the highest, with 16 and 17 common OSs in transitional and mature milk, respectively. The distribution of different oligosaccharide types in camel milk was detailed, with camel transitional milk containing 92.6% sialylated OSs, 5.9% nonfucosylated neutral OSs, and 1.5% fucosylated neutral OSs. In mature camel milk, these proportions shifted slightly to 90.0% for sialylated OSs, 9.6% for nonfucosylated neutral OSs, and 0.7% for fucosylated neutral OSs. Among the various OSs, 3′-sialyllactose (3′-SL) stood out in mature camel milk, with the highest concentration measured at 304.5 µg/mL. This detailed analysis highlights significant species-specific differences and changes over lactation stages, providing valuable insights into the composition and potential functional roles of milk OSs.

While the OS composition of camel milk has been extensively studied, the polysaccharides present in the camel milk fat globule membrane (MFGM) are a relatively unexplored area. Current research indicates that camel MFGM contains 5.90 ± 0.17 mg/g of polysaccharide, which is lower than the 8.54 ± 0.28 mg/g of polysaccharide found in bovine MFGM [74]. However, the specific types and detailed quantities of these polysaccharides have not yet been fully characterized. Future research investigating the biological functions and nutritional properties of camel MFGM polysaccharides could reveal their unique roles in health and disease, potentially leading to new nutritional insights, therapeutic applications, and the development of novel functional foods and nutraceuticals, thereby increasing the commercial value of camel milk products.

### 6.5. Minerals

The mineral composition of BC milk exhibits significant variation due to factors such as water availability, lactation period, feeding practices, season, and geographical region. Studies have identified 11 essential trace minerals in BC milk, excluding vanadium, with notable differences in the levels of certain minerals compared to those in bovine milk [30]. While some researchers have reported higher levels of calcium, phosphorus, potassium, sodium, zinc, and selenium in BC milk, others have reported lower levels of magnesium and iron [79]. Recent research by Chen et al. [80] and colleagues analyzed 17 elements across different types of milk, including cow, goat, buffalo, yak, and camel milk. Their findings revealed that the levels of arsenic, calcium, cadmium, sodium, nickel, strontium, and zinc were greater in BC milk than in other milk types. Despite numerous studies on the mineral content of Bactrian camel milk, comparisons remain challenging due to varying physiological, feeding, and environmental conditions, resulting in differing mineral composition outcomes for BC reared in different regions, particularly in China.

## 7. Bioactivities of BC Milk

Camel milk is an excellent source of essential macromolecules and contains functional compounds with antibacterial, antiviral, anti-inflammatory, antidiabetic, and anticancer properties. These beneficial components include casein, whey, oligosaccharides, and lipids. Moreover, when camel milk proteins are hydrolyzed through enzymatic or fermentation processes [81], functional peptides embedded in the primary protein structure are released [40].

### 7.1. Anti-Inflammatory Effect

Inflammation, characterized by tissue injury and intestinal microbial imbalance, is a significant cause of mortality and morbidity worldwide. Considerable evidence has demonstrated the role of camel milk in regulating inflammatory factors. In a mouse model of chronic alcoholic liver disease, camel milk regulated inflammatory cytokine production, prevented colonic dysfunction, and improved alcoholic liver injury [82]. Zhu et al. [83] reported that camel milk significantly attenuated the levels of proinflammatory cytokines (tumor necrosis factor-α, IL-10, and IL-1β) in the serum of lipopolysaccharide-induced acute respiratory distress syndrome rats. Similarly, by comparing the effects of different diets (normal diet; normal diet, then ethanol; and normal diet and camel milk, then ethanol), researchers found that camel milk reduced hepatic inflammation by downregulating the expression of inflammation-related genes (IL-1β and CXCL1) in the IL-17 pathway [84]. The beneficial effects of camel milk may be due to the combined effects of its various ingredients. Dou et al. [85] investigated the effects of camel milk whey protein (CWP) on rats with streptozotocin (STZ)-induced type 2 diabetes mellitus (T2DM) and demonstrated that CWP suppressed the inflammatory response. Additionally, Du et al. [86] reported that BRL-3A hepatocytes pretreated with CWP hydrolyzed by simulated gastrointestinal fluid exhibited resistance to heat stress-induced apoptosis via activation of the Nrf2/HO-1 signaling pathway and suppression of the NF-κB/NLRP3 axis. Fermentation is one of the most commonly used methods for liberating bioactive peptides from food matrices. A metabolomics study revealed the upregulation of beneficial metabolites after fermentation of camel milk [81]. Another study investigated the bioactive peptides from fermented camel milk using network pharmacology and molecular docking, and the results indicated that fermented camel milk exerts anti-inflammatory effects via various biological mechanisms and signaling pathways [87]. Camel milk intervention also showed anti-inflammatory effects against radiation-induced intestinal injury by decreasing the levels of proinflammatory cytokine receptors (e.g., TNF-α and IL-1β) while increasing the levels of TLR4, NF-κB, HMGB1, and IL-10, indicating that camel milk exerts anti-inflammatory effects by regulating the HMGB1/TLR4/NF-κB/MyD88 inflammatory signaling pathway [88].

The anti-inflammatory properties of camel milk and its components, such as whey protein and bioactive peptides, have been demonstrated in various studies. However, further research is needed to fully understand the underlying mechanisms and potential therapeutic applications involved. Exploring the synergistic effects of different bioactive compounds present in camel milk could provide insights into developing effective anti-inflammatory strategies. Additionally, clinical studies investigating the efficacy of camel milk or its derivatives in managing inflammatory conditions in humans would be valuable. Furthermore, investigating the potential of fermented camel milk or its bioactive peptides as functional foods or nutraceuticals could open new avenues for promoting human health and well-being.

### 7.2. Antidiabetic Effect

Type 2 diabetes mellitus (T2DM) is primarily characterized by reduced insulin sensitivity (insulin resistance) and impaired insulin secretion. Recent studies have highlighted the potential of camel milk for managing T2DM, providing evidence from various experimental and clinical investigations. Zheng et al. [89] explored the hypoglycemic effects of camel milk powder in patients with T2DM. Participants were supplemented with 10 g of camel milk powder twice daily for four weeks. The results indicated significant reductions in fasting blood glucose and 2 h postprandial blood glucose levels. Additionally, there was a notable decrease in the serum levels of resistin and lipocalin-2, adipokines that are positively correlated with diabetes. Further investigations by Han et al. [90] examined the functional impact of camel milk on glucose homeostasis, the hepatic proteome, and the phosphoproteome in high-fat diet and streptozotocin (HFD/STZ)-induced diabetic rats. Their study demonstrated that 35 days of supplementation with camel milk improved fasting glucose levels and glucose tolerance. The positive effects on lipid metabolism were attributed to the activation of AMP-activated protein kinase (AMPK). Moreover, Manaer et al. [91] evaluated the antidiabetic effects of shubat, a traditional fermented camel milk product, on streptozotocin-induced T2DM rats. Their findings showed that a high dose of shubat improved T2DM conditions by decreasing fasting blood glucose (FBG) and glycated hemoglobin (HbA1c) levels while increasing C-peptide and glucagon-like peptide-1 (GLP-1) levels. Shubat administration also protected renal function. Researchers have hypothesized that these benefits might be due to the promotion of GLP-1 release and the improvement of β-cell function. Dou et al. [85] focused on camel milk whey protein (CWP) treatment in streptozotocin (STZ)-induced T2DM rats and insulin-resistant HepG2 cell models. Intrigastric administration of CWP mitigated body weight loss and improved liver tissue structure, including cell arrangement, congestion, edema, and vacuolization. CWP feeding also upregulated the expression of insulin receptor substrate-2 (IRS-2), phosphoinositide 3-kinase (PI3K), protein kinase B (AKT), and glycogen synthase (GS). The study suggested that the hypoglycemic effect of CWP could be due to the activation of the PI3K/AKT pathway, which inhibits gluconeogenesis and promotes glycogen synthesis. In another study, Zhang et al. [40] used peptidomics to identify endogenous peptides in camel milk with dipeptidyl peptidase-IV (DPP-IV) inhibitory activity, which could contribute to its antidiabetic effects. Yu et al. [92] further analyzed the hydrolysis of camel milk proteins using 11 different proteases and assessed their antidiabetic activities through both in vitro and in vivo methods. Camel milk protein hydrolysates (CMPHs), particularly those produced with Flavorzyme, exhibited the highest α-glucosidase and DPP-IV inhibitory activities. CMPH produced with Papain demonstrated the strongest α-amylase inhibitory activity and significant proliferative effects on STZ-induced NIT-1 cells in vitro. In vivo studies revealed that CMPH–Papain was most effective at reducing fasting blood glucose, oral glucose tolerance, proinflammatory factor (IL-1β, IL-6), and triglyceride levels while increasing insulin levels in diabetic mice. Subsequent peptide identification, molecular docking, and network pharmacology analysis identified potential antidiabetic peptides from both CMPH–Flavorzyme and CMPH–Papain. Finally, Su et al. [93] identified antidiabetic peptides from camel milk protein hydrolysates, focusing on their inhibitory activities against α-amylase and DPP-IV. Camel milk proteins were hydrolyzed using Protamex and fractionated into three categories (>10 kDa, 3–10 kDa, and <3 kDa) via ultrafiltration. The <3 kDa fraction was the most effective at inhibiting enzyme activity and was further purified through gel chromatography and identified using LC–MS/MS. Among the 20 potential bioactive peptides identified, the novel peptide QEPVPDPVRGL exhibited the highest antidiabetic activity. Molecular docking revealed that this peptide interacts with α-amylase and DPP-IV through hydrogen bonds, salt bridges, and pi-alkyl interactions, effectively occupying their active sites. In summary, camel milk (CM) and its derivatives exhibit significant potential for managing T2DM through various biochemical pathways, including enzyme inhibition, β-cell function improvement, and metabolic pathway modulation. These findings provide a strong foundation for further research and the development of functional foods and nutraceuticals aimed at diabetes management.

### 7.3. Lipid-Lowering Activity

Camel milk has garnered attention for its potential lipid-lowering properties, as demonstrated through various studies employing both in vitro and in vivo models. Earlier research by Manaer et al. [91] provided foundational insights into the lipid-regulating effects of fermented camel milk. In their study involving streptozotocin-induced T2DM rats, dietary supplementation with fermented camel milk led to a marked decrease in total cholesterol, triglyceride (TG), and low-density lipoprotein cholesterol (LDL-c) while also improving high-density lipoprotein cholesterol (HDL-c) levels. The regulation of lipid metabolism observed in this study underscores the benefits of fermented camel milk as a dietary intervention for managing dyslipidemia in diabetic conditions. Ming et al. [82] conducted a significant study on the effects of camel milk on lipid accumulation in a mouse model of chronic alcoholic liver disease. Their findings indicated that camel milk effectively prevented lipid build-up in the liver, suggesting its potential as a therapeutic dietary supplement for managing lipid dysregulation associated with chronic alcohol consumption. Further investigations into the lipid-lowering effects of camel milk were carried out by Dou et al. [85], who explored the impact of camel whey protein (CWP) on T2DM rats and insulin-resistant (IR) HepG2 cell models. Their research demonstrated that CWP significantly reversed dyslipidemia in T2DM rats. In the IR-treated HepG2 cells, CWP ameliorated lipid accumulation, which points to its efficacy in improving insulin sensitivity and regulating lipid metabolism. These findings are crucial, given the strong correlation between insulin resistance, T2DM, and dyslipidemia. A study by Wang et al. [87] investigated the lipid-lowering potential of camel milk by investigating the role of bioactive peptides derived from fermented camel milk. Their research revealed that these peptides are instrumental in modulating lipid and atherosclerosis signaling pathways. The peptides’ ability to influence these pathways highlights the therapeutic potential of fermented camel milk in managing cardiovascular risk factors and lipid disorders.

Further research is warranted to elucidate the precise biochemical mechanisms involved and to assess the long-term benefits and safety of camel milk consumption in humans.

### 7.4. The Modulatory Effect of the Intestinal Microbiota

Foods can significantly influence the composition and structure of the intestinal microbiota, with changes in the abundance of certain microbial genera potentially inducing disease or providing health benefits. For example, Wang et al. [94] examined the impact of camel milk on the gut microbiota of mice, revealing a reduction in the relative abundance of *Romboutsia*, *Lactobacillus*, *Turicibacter*, and *Desulfovibrio*, while beneficial organisms such as *Allobaculum*, *Akkermansia*, and *Bifidobacterium* increased. Similarly, a study on mice with nonalcoholic fatty liver disease by Hao et al. [95] demonstrated that camel milk administration could enhance the structure and diversity of the intestinal flora by boosting beneficial bacteria and reducing harmful bacteria. Ming et al. [82] reported that camel milk mitigated intestinal microbial imbalance in a mouse model of chronic alcoholic liver disease. Furthermore, Ming et al. [84] explored the effects of different diets (normal diet; normal diet, then ethanol; and normal diet and camel milk, then ethanol) on mice with alcoholic liver disease (ALD). They found that camel milk (CM) modulates intestinal microbial communities by increasing the proportion of *Lactobacillus* and reducing the proportions of *Bacteroides, Alistipes*, and *Rikenellaceae* RC9. Moreover, researchers have investigated the effects of various thermal treatments of camel milk on the intestinal microbiota of mice. Shao et al. [96] reported that treatment of camel milk at low temperatures (65 °C) increased the relative abundance of probiotic genera such as *Akkermansia*, *Bifidobacterium*, and *Lactobacillus* while decreasing the overall diversity of the intestinal microbiota.

### 7.5. Anticancer Activity

The potential anticarcinogenic attributes of camel milk have garnered significant scientific interest, prompting numerous investigations to assess its effects in controlled in vitro experimental settings, as well as in vivo studies conducted on living organisms. A key focus of this research has been the TR35 fraction isolated from camel milk whey, which has shown promising results in the fight against cancer. A notable study by Yang et al. [97] investigated the impact of TR35 on Eca-109 esophageal cancer cells. Researchers have shown that TR35 significantly suppresses the proliferation of these cancer cells and induces apoptosis, which is the process of programmed cell death. In addition to in vitro experiments, the present study included in vivo tests in which mice with xenografted tumors were treated with TR35. Remarkably, TR35 treatment not only limited tumor growth but also did not cause weight loss in the mice, indicating that the treatment was effective and did not induce severe side effects commonly associated with cancer therapies. The benefits of camel milk extend beyond those of TR35 and its effects on esophageal cancer. Another study, conducted by Wang et al. [87], investigated bioactive peptides derived from fermented camel milk. These peptides were obtained from peptidoglycan recognition protein 1 (PGRP1) and were found to play significant roles in cancer-related signaling pathways. This discovery points to the intricate bioactivity of camel milk components and their potential to influence cancer cell behavior at the molecular level. In summary, the anticancer effects of camel milk, particularly the TR35 fraction, have been substantiated by various studies demonstrating its ability to suppress cancer cell proliferation, induce apoptosis, and alter gene and protein expression profiles. Additionally, the impact of camel milk on inflammatory pathways and the bioactivity of its peptides further underscore its potential as a complementary approach in cancer prevention and treatment. As research progresses, camel milk could emerge as a valuable natural supplement in the fight against cancer.

### 7.6. Antioxidant Activity

Several studies have highlighted the antioxidant capabilities of BC milk. For instance, a study by Chen et al. [88] showed that camel milk administration increased the survival time and rate of radiation-exposed mice, increased the levels of superoxide dismutase (SOD) and glutathione (GSH), and reduced malondialdehyde (MDA) levels, indicating its protective effects against radiation-induced damage. Further research by Ming et al. [82] revealed that camel milk could mitigate oxidative stress in mice with chronic alcoholic liver disease by enhancing the activity of antioxidant enzymes such as SOD and GSH. Similarly, Zhu et al. [83] reported that camel milk supplementation in rats with lipopolysaccharide (LPS)-induced acute respiratory distress syndrome (ARDS) alleviated the expression of oxidative stress markers in lung tissue, suggesting benefits beyond liver health. In diabetic models, camel milk has shown promising results in protecting against oxidative stress. Dou et al. [85] reported that camel milk whey protein (CWP) treatment in rats with type 2 diabetes mellitus (T2DM) induced by streptozotocin (STZ) increased the levels of superoxide dismutase (SOD) and glutathione peroxidase (GSH-Px) while decreasing malondialdehyde (MDA) levels, demonstrating its protective effects against diabetes-induced oxidative damage. A peptidomics study by Zhang et al. [40] quantified endogenous peptides from camel milk and revealed that these peptides possess antioxidative functions, further supporting the role of camel milk in mitigating oxidative stress. These findings collectively highlight the potential of camel milk and its components for regulating oxidative stress and protecting against tissue damage under various pathological conditions, including liver damage associated with T2DM and other oxidative stress-related disorders.

### 7.7. Antibacterial Activity

Compared to bovine milk, camel milk has slightly higher concentrations of certain bioactive ingredients, such as lactoferrin [98]. Lactoferrin in BC milk exhibits significant antibacterial activity, particularly at a pH range of 7.5–8.0 [99], which suggests potential applications in food preservation and clinical treatments targeting bacterial infections. Wang et al. [100] hydrolyzed camel and cow milk whey protein with trypsin, and both milk whey protein peptide fractions < 3 kDa showed the strongest antibacterial effects against Escherichia coli and Staphylococcus aureus. Camel milk whey peptides showed higher antibacterial activity, and in comparison with amino acid characteristics, both antibacterial peptide fractions exhibited high amounts of alkaline amino acids and hydrophobic amino acids.

## 8. Technological Properties

Camel milk, a newcomer to the dairy market, holds significant promise as an alternative to traditional bovine milk for meeting the growing global demand for functional food ingredients. Despite its potential for the development of new consumer products, the unique composition of camel milk presents challenges in terms of producing high-quality yogurt and cheese. To fully harness the benefits of this valuable food resource, it is essential to gain a thorough understanding of how different processing parameters—such as pH, heat treatment, high-pressure processing, microwave treatment, and homogenization—affect its physical and functional properties. These parameters play a crucial role in the production of camel milk products. Therefore, advanced processing technologies are necessary to enhance the functional properties of camel milk, unlocking its full potential for a variety of applications.

### 8.1. Buffering Capacity

The ability of milk to resist changes in pH when acids or alkalis are added or removed is due to its proteins, minerals, and organic acids [101]. This property, known as buffering capacity, varies between BC milk and bovine milk. Studies have shown distinct buffering behaviors in these two types of milk. When acid is added to camel milk, followed by a base, a loop in the pH range of 6.6 to 4.4 is observed, with the maximum buffering index occurring at approximately pH 4.4. Conversely, bovine milk exhibits a similar loop in the pH range of 5.0 to 6.6, with the highest buffering capacity occurring at approximately pH 5.1 [26]. However, different trends appear when the titration order is reversed—base followed by acid—with no loops observed and various buffering pH levels [102]. These findings suggest that camel milk has a stronger buffering capacity than bovine milk, which can be significant in the development of fermented products and human nutrition [103].

### 8.2. Effect of Heat Treatment

Camel milk, which contains essential proteins, lipids, sugars, vitamins, and minerals, is highly sought after for its rich nutrient profile. However, this nutrient richness also increases susceptibility to microbial spoilage, posing potential health risks. To address this concern, heat treatment is commonly employed to ensure microbiological safety and extend the shelf life of camel milk and its products. Nevertheless, this process can alter the sensory, physical, and chemical properties of milk, as well as degrade some of its functional components [96].

Recent research has revealed valuable insights into the effects of heat treatment on camel milk. While heat treatment may diminish the milk’s alcohol stability, it has no appreciable effect on its density, viscosity, refractive index, or pH. Pasteurization at temperatures between 60 and 75 °C did not significantly alter the protein content. However, it does lead to a notable reduction in lactose content and affects the stability of whey proteins at temperatures above 65 °C. Treating milk at 75 °C for 8–15 min can reduce the microbial load by 81.9%, with moderate impacts on protein structure, such as decreased levels of lactoferrin and albumin [98]. A study by Zhang et al. [38] examined the effects of microwave heating on camel and bovine milk and revealed that the heat resistance of whey proteins in camel milk was greater than that of proteins in bovine milk. Microwave heating between 300 and 700 W caused a reduction in κ-casein and the absence of β-lactoglobulin in camel milk, suggesting that microwave heating at 300 W for 2 min is optimal for retaining nutrients and maintaining protein stability. Proteomic analysis revealed significant changes in proteins involved in various biological processes and molecular functions in high-temperature processed liquid camel milk and camel milk powder, highlighting the impact of thermal treatment on protein functions [104]. Heat treatment also affects the formation of furosine (FRS) and 5-hydroxymethylfurfural (5-HMF), which increase significantly with increasing temperature, peaking at 135 °C. A greater degree of heat treatment also increases the aldehyde and ketone contents, affecting the flavor of camel milk [105]. Different heat treatments impact the physicochemical properties of camel milk, which in turn affects the gut microbiota in mice. Ultrahigh-temperature (UHT) treatment significantly reduces nutrient levels and alters gut microbiota diversity, whereas low-temperature, long-time (LTLT) treatment preserves more nutrients without affecting microbiota diversity [106]. Metabolomic analysis revealed significant changes in metabolites, including saccharides, glycosylamines, adenosines, and phospholipids, in raw, heated, and powdered camel milk. Thermal treatment slightly increases D-lactose and significantly increases dipeptides such as His-Pro and Lys-Trp, improving the flavor profile of camel milk [107]. Furthermore, the OS profiles of milk from dairy cows, camels, yaks, sheep, buffaloes, and horses were similar, characterized by a richness of sialylated OSs. Treatment at 65 °C had no significant effect on the concentration or distribution of OSs, whereas 135 °C heating was associated with their decrease, suggesting that more attention to temperature control is needed in milk product processing [108]. In a study by Han et al. [108], changes in the proteins of MFGM in Holstein, buffalo, yak, goat, and camel milk samples following heat treatment were investigated using an LC–MS/MS approach. The results showed that the Holstein, yak, and buffalo milk samples had similar MFGM protein components, followed by the goat and camel milk samples. Changes in lipoprotein lipase and α-lactalbumin in MFGM were dependent on the intensity of the heat treatment and were similar among the studied species, whereas changes in κ-casein, lactoferrin, and apolipoprotein A-I differed among different types of milk [109].

Overall, these studies highlight the critical balance between ensuring microbiological safety through heat treatment and preserving the nutritional and sensory qualities of camel milk. Proper heat treatment is essential to maximize the health benefits of camel milk while maintaining its safety and quality for consumption.

### 8.3. Coagulation

Milk coagulation, achievable through enzymatic methods [110] or acidic gelation [111], is essential in the production of most dairy products. However, achieving the desired gel consistency in DC milk is challenging due to its unique casein composition, micelle size, and levels of total solids, calcium, and phosphorus [112]. There are few studies on the coagulation of BC in milk. Recent studies have shown promising results with camel milk yogurt, which exhibits high pH, antioxidant activity, and crude protein content, as well as low bacterial counts and titratable acidity. However, the texture remains fragile [21]. Innovations such as the synergistic use of trisodium citrate and microbial transglutaminase are expected to enhance the gelation process by breaking down casein micelles and promoting their crosslinking into a stable gel [113]. Our recent study (unpublished) showed that camel milk yogurt could be produced with good coagulation by the addition of pectin, starch, and sodium caseinate. In the future, milk coagulation, achieved through enzymatic methods or acidic gelation, will continue to be a pivotal step in dairy product development. Advances in understanding the differences in casein composition, micelle size, total solids, calcium, and phosphorus content in dromedary camel milk will help overcome current challenges in achieving desirable gelation.

## 9. Camel Milk Products

**Figure 2:**
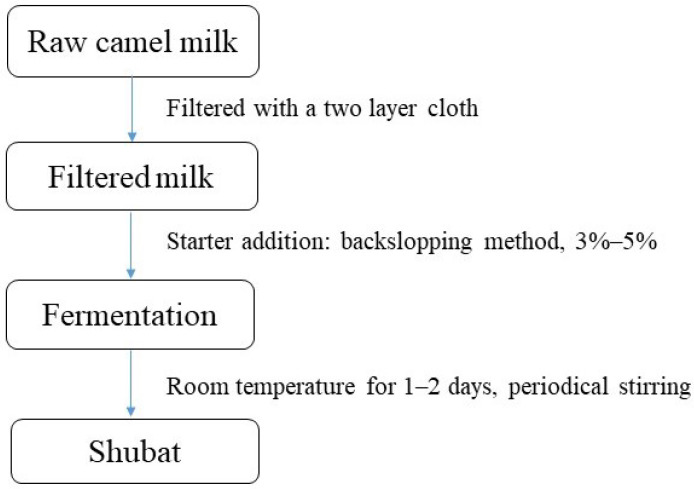
Traditional shubat processing procedure.

BC milk and its processed products are gaining increasing popularity in China because of their perceived health benefits and unique nutritional composition. While traditionally consumed fresh or fermented, modern processing techniques are enabling a wider range of BC milk products to enter the Chinese market. One of the most important camel milk products is fermented camel milk, known as shubat, which has been a staple in the diets of nomadic communities in northern China for centuries. The traditional stages of shubat processing are shown in Figure 2.

This traditional beverage is created through spontaneous fermentation by dominant lactic acid bacteria present in fresh milk [114], a technique passed through generations with minimal changes. The spontaneous fermentation of Bactrian camel milk is characterized by a dynamic shift in microbial community composition over a period of several days at ambient temperature. This process results in a significant transformation of both bacterial and fungal populations. Initially, the bacterial community is dominated by the genus *Lactococcus*, which subsequently gives way to *Lactobacillus* as the predominant genus. Concurrently, the fungal community undergoes a transition from a diverse assemblage comprising *Apiotrichum*, *Cutaneotrichosporon*, and *Candida* to one primarily dominated by *Kazachstania* and *Kluyveromyces*. The culmination of this microbial succession is the production of a distinctive fermented beverage. This end product is notable for its snow-white appearance, sour taste profile, and pleasant flavor characteristics. Moreover, the fermentation process imparts a relatively high viscosity to the final product, further distinguishing it from the original unfermented milk [115]. Recently, this traditionally prepared fermented beverage has gained popularity among all age groups in both rural and urban areas because of its reported nutritional benefits and medicinal properties, including antidiabetic, anticancer, and antituberculosis effects. However, the traditional method of spontaneous fermentation of unheated milk raises concerns about the product’s safety and potential quality issues, such as excessive sourness, creaming, and precipitation.

As awareness of these potential benefits has spread, so has the demand for BC milk and its associated products. In China, a growing number of consumers are seeking camel milk as a healthier alternative to traditional dairy products, fueling the expansion of the market and driving innovation within the industry. One notable development has been the emergence of camel milk powder, a product that has facilitated the distribution and consumption of camel milk beyond traditional production areas. By dehydrating the milk and transforming it into a shelf-stable powder, producers have been able to tap into new markets and reach consumers in urban centers across China and beyond. As the market continues to evolve, there is a growing emphasis on value-added products, such as fermented camel milk beverages, dried camel milk products, functional camel milk products, milk-based skincare and cosmetic products, and camel milk infant formula. These products not only cater to the diverse preferences of consumers but also provide opportunities for innovation and product diversification, ensuring the long-term sustainability of the industry.

## 10. Economic Potential

The economic potential of Bactrian camel (BC) milk in China is set for substantial growth, with conservative estimates forecasting a market value of approximately 2.8 billion USD by 2026. This expanding industry is driven by several critical factors, including increasing consumer awareness of BC milk’s unique properties and perceived health benefits, rising disposable incomes, evolving dietary preferences in China, and a focused interest in health and wellness due to BC milk’s rich nutritional profile and potential therapeutic properties. Additionally, as consumers seek alternatives to traditional dairy products, BC milk is positioning itself as a premium option.

Despite its current modest market share compared to conventional dairy products, BC milk’s niche status presents significant opportunities for growth and product diversification. The industry, however, faces challenges such as limited production capacity, higher price points, and the need for consumer education [116]. These challenges also signal areas for potential improvement and investment.

The development of the BC milk industry in China carries broader economic implications. It could become a significant economic driver for China’s northwestern regions, where most Bactrian camels are raised, and foster increased job creation and enhanced rural livelihoods. The industry’s growth may assist in preserving traditional herding practices and align with China’s goals for sustainable agriculture and food security. Furthermore, ongoing studies into BC milk’s nutritional and therapeutic properties are advancing scientific knowledge and potentially opening new market segments. Producers are investing in modernizing production techniques and improving supply chain efficiency to meet growing demand, while government agencies are implementing supportive policies and regulations to foster industry growth, recognizing its potential economic impact [117].

As the market matures, it has the potential not only to meet domestic demand but also to position China as a significant player in the global specialty dairy market. The unique convergence of tradition, culture, and modern innovation within the BC milk industry presents a promising economic opportunity for China, with potential positive impacts across various sectors of the economy.

## 11. Future Perspectives

BC milk possesses unique nutritional and functional properties, yet a comprehensive understanding of its composition and potential health benefits remains elusive. Extensive characterization of major and minor proteins/peptides through proteomics and peptidomics is crucial for revealing their nutritional and functional attributes. Further research on the fat composition, fatty acid profiles, distribution of different lipid classes, and role of minor lipids such as polar lipids and MFGM components in conferring health benefits is warranted, as is elucidating their cellular and molecular mechanisms. Investigating the effects of various processing techniques, such as thermal, nonthermal, fermentation, and renneting, on the composition, structure, and bioactivity of BC milk has attracted increased amounts of attention. Comprehensive studies elucidating the nutritional makeup and potential health effects of BC milk are pivotal to realizing its full potential in the dairy industry. Innovations in product development, including the use of milk powder, fermented beverages, dried products, and infant formula and the exploration of new value-added functional products, are key areas of focus. The growing Chinese market for BC milk, driven by perceived health benefits and demand for alternative dairy sources, presents opportunities for economic growth through sustainable production and commercialization of this unique dairy stream. Extensive multidimensional research coupled with product innovations tailored to consumer demands can unlock the tremendous untapped potential of BC milk as a nutritious and therapeutic food resource for the expanding Chinese market.

## 12. Conclusions

This comprehensive review highlights the unique nutritional profile and bioactive properties of Bactrian camel (BC) milk, positioning it as a promising functional food with significant potential in the Chinese market. The composition of camel milk, characterized by high protein content, distinctive fatty acid profile, and bioactive compounds, contributes to its various health benefits, including anti-inflammatory, antidiabetic, lipid-lowering, and anticancer properties. The modulatory effects on intestinal microbiota and antioxidant activities further underscore its potential as a therapeutic food.

However, the review also reveals several challenges and limitations in the current state of research. While numerous studies have explored the composition and bioactivities of BC milk, there remains a need for more comprehensive, standardized analyses across different breeds and geographical locations. The technological properties of BC milk, particularly in relation to product development, require further investigation to overcome challenges in coagulation and texture formation.

In conclusion, while BC milk shows great promise as a functional food, continued research and innovation are necessary to fully harness its potential and address current limitations in understanding and application.

## Figures and Tables

**Table 1:** Chemical composition of different milk types.

Milk Source	Alxa BC	Sonid BC	Junggar BC	DC	Cow
Total solids (%)	14.63–15.67	14.20 ± 0.01	14.85–17.49	10.80	12.40
Protein (%)	4.31–4.80	4.15 ± 0.15	3.58–3.85	3.20	3.40
Fat (%)	3.38–6.05	4.80 ± 0.14	5.37–7.44	3.10	3.70
Lactose (%)	4.56–6.23	4.90 ± 0.11	4.71–5.70	3.80	4.70
Ash (%)	0.92–0.94	0.81 ± 0.03	0.71–0.91	0.70	0.60
Na (mg/kg)	720	ND	552	474	319
P (mg/kg)	1168	ND	1068	946	901
Ca (mg/kg)	1546	1809	1442	1299	1137
K (mg/kg)	1910	1244	1449	1732	1418
Vc (mg/kg)	42.33	ND	28.23	33.00	15.00
V_A_ (mg/kg)	0.45	ND	1.11	0.27	0.38
V_B1_ (mg/kg)	0.81	ND	0.13	0.48	0.40
V_B3_ (mg/kg)	1.24	ND	ND	0.78	0.80
V_B5_ (mg/kg)	0.50	ND	ND	3.68	3.20
V_B6_ (mg/kg)	0.72	ND	0.50	0.55	0.50
V_E_ (mg/kg)	2.73	ND	1.43	0.02	1.00
References	[19,26,27,28]	[27,29]	[18,30,31,32,33]	[34,35,36]	[33,34,35,37]

Abbreviations: ND, not determined; BC, Bactrian camel; DC, Dromedary camel.

## Data Availability

Data are contained within the article.
